# Ingestion of Cylindrical Battery and Medical Treatment

**DOI:** 10.12669/pjms.37.2.3225

**Published:** 2021

**Authors:** Mehmet Nuri Kosar, Ozkan Gorgulu

**Affiliations:** 1 Mehmet Nuri Kosar Department of General Surgery, University of Health Sciences, Antalya Training and Research Hospital, Antalya, Turkey; 2 Ozkan Gorgulu Department of Anesthesiology and Reanimation, University of Health Sciences, Antalya Training and Research Hospital, Antalya, Turkey

**Keywords:** Cylindrical AA battery, Digestive system, Medical treatment, Abdominal radiography

## Abstract

Due to their chemical properties, accidental or suicidal ingestion of batteries into the digestive system can cause fatal complications; Treatment should not be delayed and close monitoring is required. A 26-year-old male patient is treated by the psychiatry department with diagnoses of antisocial personality disorder and depressive adjustment disorder. He consulted with the complaint of ingesting cylindrical AA battery for suicidal purpose. In our case, the cylindrical AA battery in the duodenum was removed from the rectum at the end of the third day without any complications. However, the continuous movement of the cylindrical AA battery with lactulose treatment in the gastrointestinal tract and the support of this movement with abdominal radiographs can reduce the risk of fatal complications. When planning the battery treatment in the gastrointestinal tract, the location of the battery and whether it is mobile should be determined. While obstruction of oesophagus by batteries requires emergency surgical treatment, batteries that remained fixed in the stomach for longer than 48 hours need to be treated with surgical or endoscopic methods.

## INTRODUCTION

Whereas foreign body ingestion is more common in pediatric patients^1^ it can also be observed due to involuntary reasons^2^,^3^ or due to psychiatric disorders. Among psychiatric disorders, diseases like pica type eating disorders which cause iron deficiency anemia that are frequently seen in childhood period are observed. But in the case of adults, generally in imprisoned patients with accompanying psychiatric disorders, suicidal attempts by ingesting cylindrical AA batteries aiming at (a variety of) positive gains are observed. Dunphy L et al. reported the medical and surgical treatment of the two repeated attempts of ingesting 8 and 7 AA (standard size single cell cylindrical dry battery) batteries of an incarcerated patient of age 37 with dissocial, paranoid, borderline and emotionally unstable personality disorder.^4^ Non-chemical foreign bodies are usually harmless; however, batteries, when ingested, cause very severe and fatal complications in the digestive system due to their chemical components. Here we are presenting a case who ingested an AA battery for suicidal purpose.

## CASE REPORT

**Fig.1:**
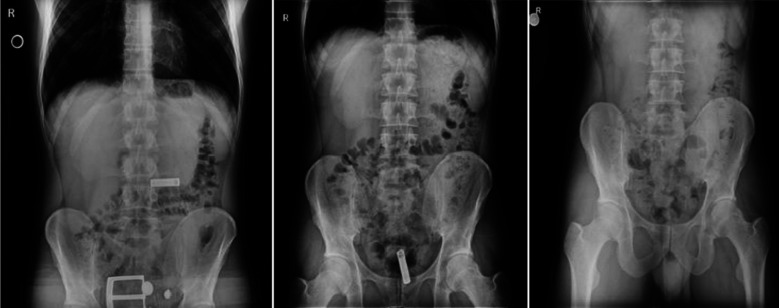
The course of the battery with direct abdominal X-rays.

**Fig.2:**
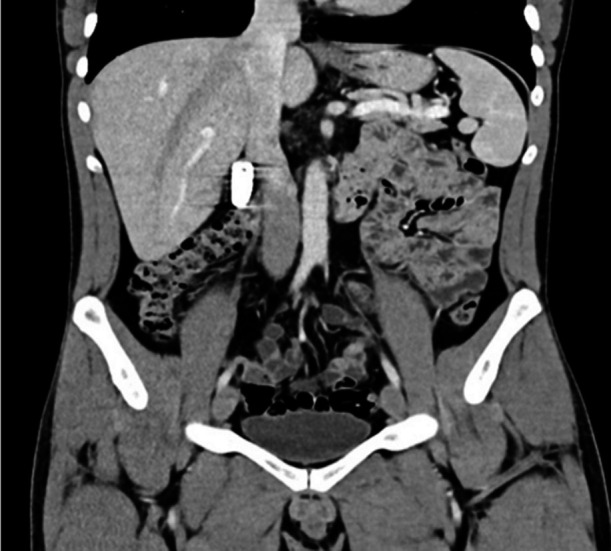
Computer Tomography (CT) image of battery in the 2nd part of the duodenum.

A 26-year-old male patient who has been imprisoned for two years and has no systemic disease is treated by the psychiatry department with diagnoses of antisocial personality disorder and depressive adjustment disorder. On the 5th day of September 2019, he consulted with the complaint of AA battery ingestion for suicidal purpose three hours before applying to the hospital. Having ingested an AA battery and razor blade for suicidal purpose one year ago, the patient was subjected to emergency surgical intervention at another hospital, and the foreign bodies were removed from the digestive tract with laparotomy. Psychiatry consultation ended with the result that the patient has no Pica associated symptoms/conditions, he does not have anemia and that with the aim of getting away of prison conditions for a temporary period of time, with his depressive mood he ingested the only foreign object he could find, the batteries. At the time of his examination at the emergency room, his general condition was good, his vital findings were stable, and his laboratory findings were normal. Direct radiological examination in the emergency room revealed that the AA battery crossed the first continent of the pylorus and duodenum and stopped transversely in the third continent (X-ray: Fig.1). Abdominal tomography was performed in order to determine the exact intestinal location of the battery and it was confirmed that it passed to the distal of the pylorus (CT: Fig.2).

The patient who was kept under observation at the emergency room was per orally administered 45ml/30 g lactulose as a single dose per day for three days. The patient was hydrated with 100 ml h^-1^ Ringer’s lactate solution and his pro-kinetic (lactulose) treatment was continued. The battery was visualized in the ileocecal junction in the abdominal x-ray taken on the 2nd day.

The battery was visualized in the rectum in the abdominal x-ray taken early on the 3rd day (Fig.1). In the evening on the 3rd day, the battery was removed from the body by rectal defecation (Fig.1). The patient was discharged as no abnormal findings were observed in his general condition and abdominal examination. No deformation of the cylinder AA battery was observed.

## DISCUSSION

Button battery and pediatric patients are more commonly observed in the literature^1^, but there are patient groups that have ingested cylindrical AA battery as in our case, although rare. Complications such as perforation^3^ and fatal haemorrhage^5^ may develop when battery is ingested as a foreign body into the gastrointestinal systems due to its chemical composition. As for battery ingestion, the American Society for Gastrointestinal Endoscopy Guidelines recommend emergency treatment in case of esophageal obstruction, whereas cylindrical batteries should be removed in the event that they remain in the stomach for more than 48 hours.^3^,^6^ This period is indicated as 24 hours for sharp foreign bodies such as nails in the upper gastrointestinal system.^7^ In our case, cylindrical AA battery being on constant move in the gastrointestinal system with the lactulose treatment and the reinforcement of this movement with abdominal radiography might have reduced the risk of fatal complication. The first issue to be addressed here is whether the cylindrical AA battery has caused esophageal obstruction, and the second one is whether the cylindrical AA battery has made its passage from stomach to duodenum. The distinction between surgical and endoscopic or medical treatment should be made at this point. According to Hammami MB et al., for the patient who ingested two AA batteries, endoscopic removal was applied for the battery which was in the stomach, whereas for the one which passed to the duodenum a daily medical treatment with 17 g polyethylene glycol 3350 pro-kinetic agent was applied and the patient ejected the second battery at the seventh day by defacation.^8^ In our case, with no oesophageal obstruction, although he had laparotomy previously, we chose medical treatment method as the cylindrical AA battery remained in the stomach for less than 1 day.

## CONCLUSION

Batteries may lead to fatal complications in the gastrointestinal system. Battery remaining for longer periods in the oesophagus and stomach increases the risk of complications. Whether to implement invasive or medical methods as treatment can be decided depending on battery’s movement. The medical approach is possible through close monitoring and serial radiography in patients who ingested cylindrical battery, including those who had laparotomy or intestinal surgery.

### Author’s Contribution:

**MNK:** Did manuscript writing, edited the manuscript.

**OG:** Reviewed the manuscript, gave final approval.

**MNK:** Responsible and accountable for the accuracy or integrity of this study.
